# Clinical Study of the Relation between Mucosal Healing and Long-Term Outcomes in Ulcerative Colitis

**DOI:** 10.1155/2013/192794

**Published:** 2013-05-09

**Authors:** Kaoru Yokoyama, Kiyonori Kobayashi, Miyuki Mukae, Miwa Sada, Wasaburo Koizumi

**Affiliations:** Department of Gastroenterology, Kitasato University School of Medicine, 2-1-1 Asamizodai, Minami, Sagamihara 252-0380, Japan

## Abstract

*Background and Objectives.* Mucosal healing (MH) is considered an important therapeutic goal in ulcerative colitis (UC). We evaluate the severity of intestinal inflammation and clarify the relation between MH and long-term outcomes. *Methods.* The study group comprised 38 patients with UC in clinical remission on total colonoscopy who were followed up for at least 5 years. Clinical remission was defined as a Mayo score of 0 for both stool frequency and rectal bleeding. Colonoscopic findings were evaluated into 4 grades according to the Mayo endoscopic subscore (MES). *Results.* During clinical remission, the MES was 0 in only 24% of the patients, 1 in 40%, 2 in 26%, and 3 in 10%. Seventy-six percent of the patients thus had active disease on colonoscopy. After initial colonoscopy, the cumulative rate of remission maintenance was 100% in MES 0, 1 in 93%, 2 in 70%, and 3 in 50% at 6 months and 78%, 40%, 10%, and 0%, respectively, at 5 years (*P* < 0.001). *Conclusion.* Many patients with UC in clinical remission have active lesions. Patients with a higher MES have a higher rate of recurrence. To improve long-term outcomes, an MES of 0 should be the treatment goal.

## 1. Introduction

The treatment response and prognosis of inflammatory bowel disease (IBD) have been evaluated on the basis of clinical remission, dose reduction of steroids, avoidance of surgery, and other factors. However, since the advent of potent drugs such as antitumor necrosis factor-*α* monoclonal antibody preparations, treatment response has been assessed on the basis of “mucosal healing” as evaluated on colonoscopy [1–3]. However, standard criteria for the evaluation of disease severity and definitions of mucosal healing on colonoscopy are currently unavailable [4]. Moreover, long-term studies examining whether mucosal healing actually contributes to remission maintenance in IBD are scant. 

We performed colonoscopy in patients with ulcerative colitis (UC) in clinical remission to evaluate the presence or absence of intestinal inflammation and to retrospectively analyze the relation between mucosal healing and long-term outcomes during 5 years of followup.

## 2. Methods

### 2.1. Patients

 Among 46 patients with ulcerative colitis in clinical remission who underwent colonoscopy in our hospital from January 2005 through December 2006, we studied 38 patients in whom the severity of intestinal inflammation was evaluated on total endoscopy; all patients were followed up for at least 5 years. Eight patients who received corticosteroids at the time of colonoscopy were excluded from the study because steroids can mask clinical signs and symptoms. 

As for the demographic characteristics of the patients, there were 18 males and 20 females. The mean age at the onset of UC was 38.8 ± 13.0 years (range, 16 to 73). The mean disease duration at colonoscopy was 15.8 ± 9.0 years (range, 1 to 37). The disease type was flare-ups and remission in 37 patients and initial attacks in 1 (Table 1). Disease extent at the onset of UC was pancolitis in 11 patients (29%), left-sided colitis in 11 (29%), proctitis in 11 (29%), and unknown in 5 patients (13%) who were initially treated at other hospitals. Overall, 13 patients (34%) had previously received treatment during hospitalization. 

At the time of colonoscopy, 36 patients (95%) were receiving drug therapy, which included 5-aminosalicylic acid (5-ASA) in 34 patients (94%), immunomodulators in 1 (3%), and local therapy such as 5-ASA and steroid suppositories and enemas in 7 (19%) (some overlap). 

### 2.2. Evaluation of UC Activity

The Mayo score [5] was used to evaluate UC activity. The Mayo score consists of 4 components: stool frequency, rectal bleeding, endoscopy findings, and physician's global assessment. Each component is assigned a score of 0 to 3; the total score ranges from 0 to 12. The higher the score, the higher is the disease severity. In the present study, clinical remission was defined as a score of 0 for both stool frequency and rectal bleeding. Flare-ups were defined as the need for additional drugs or modification of the treatment regimen because of exacerbation of clinical symptoms. 

### 2.3. Colonoscopic Examination and Evaluation of Intestinal Inflammation

Before colonoscopic examination, all patients were provided with a detailed explanation of the examination objectives and gave written informed consent. An oral polyethylene glycol lavage solution was used for bowel preparation. An anticholinergic agent (scopolamine butylbromide 10 mg) or glucagon (1 mg) was given intramuscularly as premedication. A small-bore, high-magnification colonoscope (PCF-Q240ZI, Olympus Co. Ltd., Tokyo, Japan) was used. All examinations were performed by 3 endoscopists specialized in the diagnosis and treatment of IBD who each had at least 15 years of experience in colonoscopy. 

**Figure 1:**
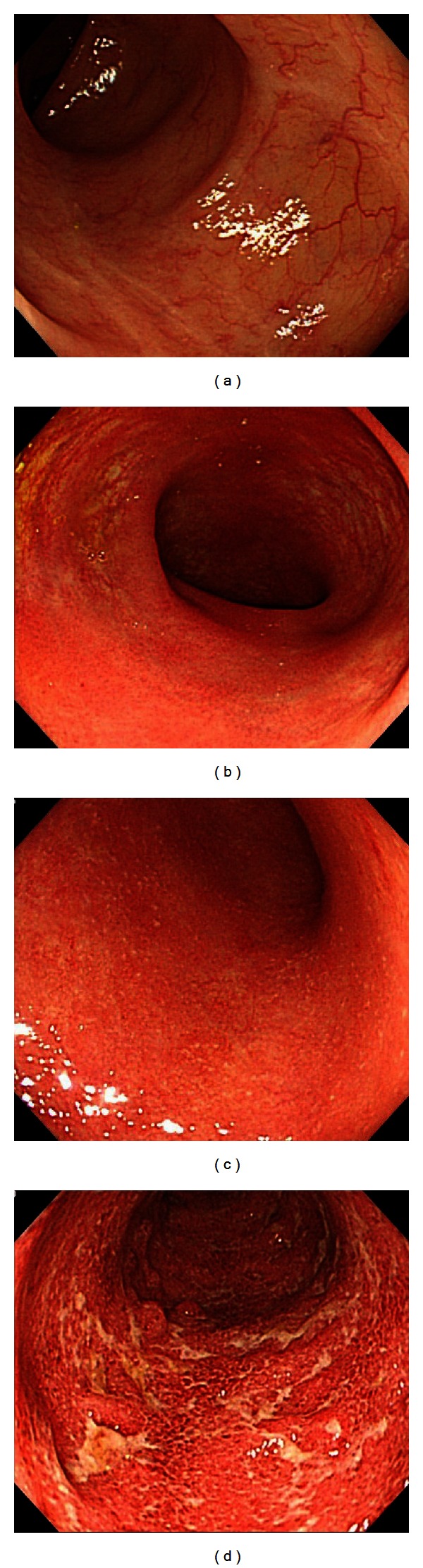
(a) An endoscopic image of the colonic mucosa, a Mayo endoscopic subscore of 0. The mucosal has a visible vascular pattern, and scattered white ulcer scars are evident. (b) An endoscopic image of the colonic mucosa, a Mayo endoscopic subscore of 1. The vascular pattern of the mucosa is decreased, with mild erythema. (c) An endoscopic image of the colonic mucosa, a Mayo endoscopic subscore of 2. The vascular pattern of the mucosa is decreased, with marked erythema and multiple erosions. (d) An endoscopic image of the colonic mucosa, a Mayo endoscopic subscore of 3. The mucosa shows marked erythema and ulcers.

The Mayo endoscopic subscore (MES) was used to evaluate the severity of intestinal inflammation on colonoscopy [5] as follows: MES 0, normal or inactive disease; MES 1, mild disease (erythema, decreased vascular pattern, and mild friability); MES 2, moderate disease (marked erythema, absent vascular pattern, friability, and erosions); and MES 3, severe disease (spontaneous bleeding and ulcerations) (Figures 1(a) to 1(d)). The entire colon was observed by white-light colonoscopy, and sites with the most severe inflammation were evaluated.

### 2.4. Study Variables

The severity of intestinal inflammation was evaluated on colonoscopy during clinical remission of disease. The relation between the severity of colonoscopic findings and the maintenance of clinical remission was then studied up to 5 years after initial colonoscopy. The study was approved by the ethics review committee of our hospital. 

### 2.5. Statistical Analysis

Data are presented as means ± standard deviation. For statistical analysis, the chi-square test and Fisher's exact test were used to compare incidence rates. One-way factorial analysis of variance and Scheffe's test were used to compare parametric variables between multiple unpaired groups. The Kruskal-Wallis test was used to compare nonparametric variables among multiple groups. Cumulative rates of remission maintenance were calculated using the Kaplan-Meier method. Log-rank tests (Peto & Peto modification) were used to determine whether differences among multiple groups were statistically significant. *P* values of less than 0.05 were considered to indicate statistical significance. Statistical analyses were carried out using StatMate IV software, version 4.01 for Windows (ATMS, Tokyo, Japan). 

## 3. Results

### 3.1. Mucosal Healing Rates

The MES at initial colonoscopy during clinical remission was 0 in 9 patients (group A), 1 in 15 (group B), 2 in 10 (group C), and 3 in 4 (group D) (Table 2). Active intestinal lesions were found in 29 patients in clinical remission (76%), despite no diarrhea or bloody stools. Demographic characteristics such as disease duration and disease extent did not differ significantly among the 4 groups. Remission induction regimens at the time of flare-ups before the most recent colonoscopic examination and the duration of remission maintenance up to the time of colonoscopy also did not differ among the 4 groups. There was also no difference in drug therapy at the time of initial colonoscopy. 

### 3.2. Cumulative Rates of Remission Maintenance after Colonoscopy

**Figure 2:**
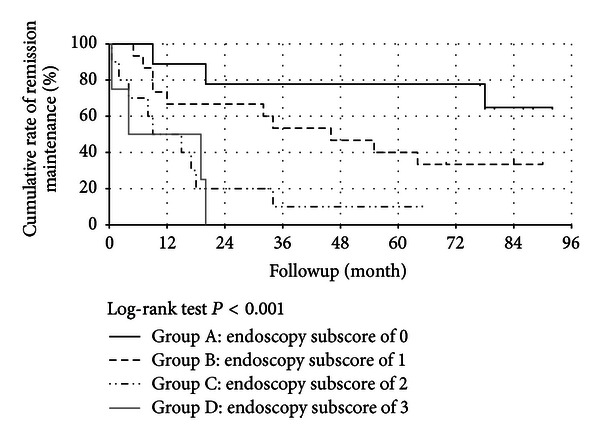
Cumulative rates of remission maintenance according to Mayo endoscopic subscore.

 The cumulative rate of remission maintenance 6 months after initial colonoscopy as evaluated by the Kaplan-Meier method was 100% in group A, 93% in group B, 70% in group C, and 50% in group D. At 2 years the cumulative rates of remission maintenance were 78%, 67%, 20%, and 0%, respectively, and at 5 years the rates were 78%, 40%, 10%, and 0%, respectively. The cumulative rate of remission maintenance differed significantly among the 4 groups (multiple log-rank test (Peto & Peto modification), *P* < 0.001) (Figure 2). Regimens during followup after colonoscopy did not differ among the 4 groups (Table 2). Adherence to drug therapy was good. At the time of flare-ups, 1 patient in group B and 1 in group D were admitted to receive treatment. 

## 4. Discussion

UC is a chronic IBD that develops in relatively young adults and requires long-term, continuous drug therapy to stabilize disease. However, a substantial number of patients are unable to strictly comply with drug therapy during followup, increasing the risk of relapse [6]. In fact, some patients request dose reduction or treatment withdrawal soon after the resolution of symptoms such as bloody stools and abdominal pain. A considerable number of patients thus do not take medication as directed. Not only patents, but also some physicians instruct patients to decrease the dose or discontinue drug therapy soon after the resolution of symptoms, often leading to relapse. In Japan, the number of patients with UC has been increasing annually. We speculate that increasing numbers of patients with UC are being treated by not only IBD specialists, but also general internists. Even if patients with active UC are initially treated by IBD specialists, maintenance therapy after remission induction is probably often entrusted to general internists. 

Colonoscopy is essential for an in-depth assessment of intestinal inflammation in UC. Recent studies have reported that narrow band imaging (NBI) and magnifying endoscopy in addition to white-light endoscopy are useful for detailed assessment of the mucosa and facilitate evaluation of the severity of intestinal inflammation. These techniques are also useful for predicting outcomes, including the risk of recurrence [7, 8]. However, NBI and magnifying endoscopy can be routinely performed in only a limited number of hospitals. In general hospitals, the severity of intestinal inflammation is evaluated, and the treatment policy is decided on the basis of white-light colonoscopic findings. We therefore studied variables that can be evaluated on white-light colonoscopy. Moreover, the inclusion of many variables in the colonoscopic evaluation of the severity of intestinal inflammation in UC leads to complexity, as well as considerable variation in the results of evaluation among endoscopists [9]. In the present study, we therefore used the MES [5], a relatively straightforward evaluation system that has been widely used in clinical trials of new drugs and other clinical studies in patients with UC. To ensure that lesions were accurately diagnosed, the following precautions were taken. Because small lesions are difficult to diagnosis after inadequate intestinal lavage, all patients received pretreatment with oral intestinal lavage solution (polyethylene glycol). The same model of colonoscope was used in all patients to examine and evaluate the entire colorectum. Colonoscopy was performed by specialists of IBD who had at least 15 years experience in colonoscopy. In addition, colonoscopic findings were evaluated by endoscopists who were blinded to the patients' outcomes. 

Our results showed that about three-fourths of all patients continued to have active intestinal inflammation even during clinical remission of UC. All patients received drug therapy to induce remission at the time of flare-ups before the most recent colonoscopic examination. The treatment regimens did not differ among the 4 groups. The duration of clinical remission before colonoscopy also did not differ significantly among the 4 groups. In addition, therapy being received by patients at the time of colonoscopy was similar in the 4 groups. However, the 4 groups had different severities of intestinal inflammation on colonoscopy, ranging from an MES of 0 to 3. Intestinal lesions thus did not resolve in many patients, despite the clinical remission of disease. Our results support the importance of evaluating the degree of mucosal healing on colonoscopy. Frequent divergence between clinical remission and the remission of intestinal lesions in UC has also been confirmed in previous prospective studies [10, 11]. 

Many previous studies evaluating the effects of various types of treatment on remission maintenance in UC were relatively short (about 1 year) [10, 11]. In contrast, we analyzed long-term outcomes over the course of 5 years by assessing the severity of intestinal lesions on colonoscopy. Relapse occurred within 5 years in nearly all patients who were in clinical remission, but had erosions and ulcers with an MES of 2 or 3 on colonoscopy. Even among patients with an MES of 1 who had erythema and decreased vascular pattern on colonoscopy, the rate of remission maintenance at 5 years was only 40%. In contrast, the rate of long-term remission maintenance was 78% in patients whose condition improved to an MES of 0 with no evidence of inflammation on colonoscopy. The rate of remission maintenance in UC may be related to the remission maintenance regimens given during followup and adherence to drug therapy [6]. In our study, the regimens given after colonoscopy did not differ from those used at the time of colonoscopy, and adherence to treatment was good. Because patients with active lesions did not have symptoms at the time of colonoscopy, some patients did not perceive the need for, or agree to receive, more aggressive therapy, but adherence with the treatment regimen being used was good. From 2005 through 2006, when initial colonoscopy was performed in this study, treatment with antitumor necrosis factor-*α* monoclonal antibody preparations was not covered by the National Health Insurance in Japan. In the latter half of 2006, treatment with azathioprine, an immunomodulator, finally became eligible for reimbursement by the National Health Insurance. Owing to these factors, patients continued to receive generally the same regimens during followup as those received at the time of initial colonoscopy. The differences in the remission maintenance rates among the 4 groups are therefore most likely attributed to differences in the severity of intestinal inflammation at the time of colonoscopy and not to differences in or modifications of treatment regimens. Therefore, we believe that the treatment goal should be an MES of 0. 

 However, many previous clinical studies defined mucosal healing as an MES of 0 or 1 [10, 11]. One study evaluating the effectiveness of mesalazine for remission maintenance reported no difference in the rate of relapse at 1 year between patients with an MES of 0 and those with an MES of 1 [10]. In a prospective study assessing the effectiveness of infliximab for remission maintenance, the symptomatic remission rate after 54 weeks was 73% in patients with an MES of 0 on colonoscopy performed after remission induction and 47% in those with an MES of 1, indicating that more than half of the latter patients had relapse. However, among patients who had clinical remission 8 weeks after treatment with infliximab, there was no difference in clinical outcomes (including colectomy) at 54 weeks between patients with an MES of 0 and those with an MES of 1 at 8 weeks [11]. In the ACT 1 trial [1], the rate of steroid-free remission after 54 weeks was 63% in patients with an MES of 0 on colonoscopy after 8 weeks of treatment, as compared with only 46% in patients with an MES of 1. Whether a difference between an MES of 0 and an MES of 1 influences clinical outcomes thus remains controversial. In addition, these findings were obtained from studies with relatively short follow-up periods of about 1 year. In our study, the cumulative remission maintenance rate 6 months after colonoscopy was similar in group A (MES 0, 100%) and in group B (MES 1, 93%). However, the cumulative remission maintenance rate was 78% in group A and 67% in group B at 2 years and 78% in group A and 40% in group B at 5 years, indicating that the cumulative remission maintenance rate decreased with time in patients who had an MES of 1; moreover, the divergence between group A and group B increased. These findings also support the notion that an MES of 0 should be the goal of treatment. Indeed, it may be difficult to accurately assess colonoscopic findings and in particular to distinguish between an MES of 1, indicating mild disease, and an MES of 0, indicating normal or inactive disease [9]. Efforts should be made, including adequate pretreatment, to ensure that evaluations can be performed as accurately as possible. Because our study was conducted in a single hospital, differences in the conditions of endoscopic examination and in the assessment of endoscopic findings were most likely minimal. However, further multicenter, prospective studies of larger numbers of patients are needed to confirm our findings and to establish the clinical positioning of an MES of 0 and an MES of 1. 

As mentioned above, even patients with UC in clinical remission who have active lesions on colonoscopy have a high rate of relapse during followup. To achieve remission maintenance, the goal of therapy should be to downgrade inflammation in patients who have intestinal inflammation. Physicians should recognize that intestinal inflammation accompanied by findings such as erythema and erosions persists after the resolution of symptoms in an appreciable number of patients. Education of patients is also important. Patients should be advised not to stop drug therapy on their own initiative, even if clinical symptoms resolve. 

 Substantial progress has been made in drug therapy for UC. Antitumor necrosis factor-*α* monoclonal antibody preparations, potent immunosuppressants such as tacrolimus and cyclosporine, and new treatments such as cytapheresis are now available to complement conventional preparations such as 5-ASA and steroids. Consequently, mucosal healing can now be achieved in increasing numbers of patients. Immunomodulators such as azathioprine and 6-mercaptopurine are widely known to be effective as remission maintenance therapy. Further prospective studies are needed to examine the relation between mucosal healing and long-term remission maintenance for each of these newer treatments. 

We evaluated the effects of mucosal healing on long-term outcomes in patients with UC. However, our study had several limitations: it was conducted in a single center and had a small sample size. Further multicenter prospective studies in larger numbers of patients are needed to confirm our findings and to further delineate the clinical significance of mucosal healing in UC. 

## Figures and Tables

**Table 1:** Demographic characteristics of patients.

No. of patients	38
Sex	
Male	18 (47%)
Female	20 (53%)
Age at disease onset (range)	38.8 ± 13.0 years (16–73)
Disease duration (range)	15.8 ± 9.0 years (1–37)
Disease type	
Flare-ups and remission	37 (97%)
Initial attacks	1 (2%)
Lesion extent at disease onset	
Pancolitis	11 (29%)
Left-sided colitis	11 (29%)
Proctitis	11 (29%)
Unknown	5 (13%)
History of treatment in hospital	
Yes	13 (34%)
No	25 (66%)
Drug therapy at colonoscopy	
Present	36 (95%)
5-Aminosalicylic acid	34 (94%)
Immunomodulators	1 (3%)
Local therapy	7 (19%)
None	2 (5%)

**Table 2:** Treatment regimens according to the Mayo endoscopic subscore.

Group	A	B	C	D
Endoscopic subscore	0	1	2	3
No. of patients (%)	9 (24%)	15 (40%)	10 (26%)	4 (10%)
History of treatment in hospital				
Yes	4 (44%)	5 (33%)	3 (30%)	1 (25%)
No	5 (56%)	10 (77%)	7 (70%)	3 (75%)
Remission induction therapy before colonoscopy				
Present	9 (100%)	15 (100%)	10 (100%)	4 (100%)
5-Aminosalicylic acid	9 (100%)	15 (100%)	10 (100%)	3 (75%)
Corticosteroid	3 (33%)	4 (27%)	2 (20%)	1 (25%)
Local therapy	5 (56%)	7 (47%)	6 (60%)	3 (75%)
Duration of remission maintenance before colonoscopy (months)	36.1 ± 32.4	28.7 ± 26.9	21.2 ± 22.4	24.3 ± 9.8
Drug therapy at colonoscopy				
Present	8 (89%)	14 (93%)	10 (100%)	4 (100%)
5-Aminosalicylic acid	8 (89%)	13 (87%)	10 (100%)	3 (75%)
Immunomodulators	1 (11%)	—	—	—
Local therapy	1 (11%)	2 (14%)	3 (30%)	1 (25%)
None	1 (11%)	1 (7%)	—	—
Treatment after colonoscopy				
Present	8 (89%)	11 (73%)	10 (100%)	4 (100%)
5-Aminosalicylic acid	8 (89%)	10 (77%)	10 (100%)	3 (75%)
Immunomodulators	—	—	—	—
Local therapy	—	1 (7%)	2 (20%)	1 (25%)
None	1 (11%)	4 (27%)	—	—
